# Focus on sex differences in grant applications submitted to the Netherlands Organization for Health Research and Development

**DOI:** 10.1186/1475-9276-6-13

**Published:** 2007-10-24

**Authors:** Debby G Keuken, Joke A Haafkens, Niek S Klazinga

**Affiliations:** 1 Department of General Practice, Academic Medical Center-University of Amsterdam, Division of Clinical Methods and Public Health, Meibergdreef 15, 1105 AZ Amsterdam, The Netherlands; 2 Department of Social Medicine, Academic Medical Center-University of Amsterdam, Division of Clinical Methods and Public Health, Meibergdreef 15, 1105 AZ Amsterdam, The Netherlands

## Abstract

**Background:**

Several measures have been implemented at international level to ensure that there is a greater focus on sex differences in health research. This study evaluates the effect of various formal incentives that were introduced by a Dutch financer of health research to encourage applicants to include sex differences in research proposals.

**Methods:**

We sampled 213 health research proposals submitted in 2003 to the programmes Prevention (N = 104) and Innovation (N = 109) by the Netherlands Organization for Health Research and Development (ZonMw). These proposals were analysed and categorized with regard to the expressed intention to take sex differences into consideration. Furthermore, those proposals in which such intention was absent were appraised by researchers to determine whether an intention of this kind would have been relevant.

**Results:**

We found that 23 % of proposals submitted to Prevention (incentive: programme specific instructions) and 10% of those submitted to Innovation (general set of guidelines) took account of sex differences (difference 13%; 95% CI: 3.1–22.9). Conversely, 66% of the research proposals in Prevention, and 20% in Innovation, failed to take sex differences into consideration, even though this might well have been relevant.

**Conclusion:**

There is still insufficient incentive for those submitting research proposals to ZonMw to systematically incorporate sex differences when drafting such documents. The provisions in ZonMw's policy need to be amended and better monitored. For this, we formulated some recommendations.

## Background

Health research is the basis of optimal health care delivery. Public organizations for the funding of health research play a key role in supporting this research. In the past, women were often under-represented among the subjects participating in clinical health research studies [1]. This was based on the belief that, aside from the reproductive system, males and females had basically the same biology. Another factor was a system of research ethics which sought to protect female test subjects of childbearing age from harm, should they become pregnant. In the 1980s, scientists and women's health activists, particularly in North America, began to express concerns about this approach to research. It was feared that the under-representation of women in clinical research would hamper an accurate understanding of the impact of biological sex factors or socially constructed gender factors on health and disease, and that this might lead to less appropriate health care delivery for both sexes [1].

In several countries, public organizations for the funding of health research have responded to this situation by adapting their policies to take account of these concerns. In 1993, the National Institutes of Health (NIH) Revitalization Act was approved by the US Congress. Since then, the NIH has required that men, women and minorities should be adequately represented in clinical studies. It further stipulates that research designs should allow valid and meaningful analysis of differences between the sexes [2].

In 1997, following the adoption of a general policy to improve women's health, Health Canada introduced a guideline for drug research and registration. The aim was to encourage the inclusion of women at all stages of drug development, and to facilitate the detection of significant sex-related differences in drug response [3].

The revised NIH policy has been evaluated by studies carried out both by the United States General Accounting Office (GAO) and by independent researchers. These studies suggest that, while progress has indeed been made in the recruitment of women for NIH-funded research, research reports often fail to include an analysis of data by sex [4-6]. Marrocco et al. evaluated the intentions of Canadian clinical investigators to recruit individuals of both sexes and to analyse data by sex. This involved an examination of research ethics applications at a Canadian health sciences centre over a five-year period (1995–2000). Their study revealed that 97.6% of researchers working on non-sex-specific conditions intended to recruit both men and women, while only 20.2% planned to perform analyses of data by sex [7].

In 2000, the European parliament also made a clear commitment to promote gender equality in EU-funded research. The aim was to achieve a balanced participation of male and female scientists in projects (40% women), while ensuring that studies focus on sex and gender related factors [8]. Researchers participating in the 6th Framework Programme (FP6), which runs from 2002 to 2007, were asked to describe and justify the composition of their study populations according to sex. They were also required to indicate how they plan to integrate a focus on sex and gender issues, where appropriate, into the objectives and methodology of their research proposals. They were also asked to provide a gender action plan [9]. Both these measures and the gender action plans are currently the subject of monitoring studies, the results of which will be made available following the completion of FP6 in 2007.

In his study of international health research policies promoting sensitivity to sex or gender differences, Caron noted that national health research funding organizations in various European countries have also undertaken initiatives to promote greater sensitivity to sex differences in health research. No assessment of these initiatives, in the form of published studies, is yet available [10].

The Netherlands Organization for Health Research and Development (ZonMw) is one of the above-mentioned organizations. ZonMw is the main source of government funding for health research in the Netherlands. It funds a broad range of health research, both basic and applied, as well as research into the implementation of health care improvements. To this end, it has many different grant programmes, each with its own research focus and set of priorities [11]. In 1999, ZonMw adopted the general policy that its financial support is subject to the provision that studies must give sufficient emphasis to diversity factors, such as sex, age and ethnicity [12]. The organization implemented this new policy in two different ways. All those putting forward and assessing grant proposals were provided with a set of guidelines. These contained general information about ZonMw's commitment to an adequate focus on diversity factors in health research. Furthermore, within several grant programmes, changes were also made to the instructions for grant applicants (and for those reviewing grant applications). These modifications involved the inclusion of specific questions on diversity issues.

The aim of the present study is to evaluate the success of this diversity policy in encouraging applicants to take sex differences into consideration when drafting research proposals.

To this end, we reviewed various research proposals that were submitted to two ZonMw programmes in 2003. The programmes in question were Innovative Research (Innovation) and Prevention in Health Care (Prevention). Applicants to the first of these programmes were only given general information about ZonMw's commitment to ensuring that due consideration is given to diversity factors in health research. In the instructions accompanying the application form, those applying to the second programme were specifically asked if they were aware of any relevant sex, age or cultural differences with respect to their study's target population. If this was the case, they were asked how the study would take these differences into consideration (see table 1).

**Table 1:** Summary of guidelines regarding the consideration of diversity factors for grant proposals of two Programmes of ZonMw: Innovation and Prevention.

**General instructions of ZonMw for writing grant proposals**	
Relevance	
"*ZonMw has a number of general spear-points: sex (gender differences), culture (cultural differences in prevention and care for citizens from a variety of backgrounds), age (extra focus on young people and the elderly) and the point-of-view of patients/consumers (cooperation with the ultimate target group). These factors are specifically involved in the assessment, serving as relevance criteria. The grant applications should contain an adequate explanation of the reasons for including the aspects in question in the study, or for omitting them, as the case may be. In projects where this is relevant, the process of quality assurance (..) will include a check to determine whether these factors have been adequately fleshed out in the project plan*."	
Quality.	
*Under the heading of "Quality", certain constraints are imposed on the content of the Action Plan: "Where appropriate, the Action Plan also gives details of the way in which the factors of gender, age, cultural background and/or other relevant characteristics that form an essential part of the objective, have been fleshed out*....".	
**Programme Prevention ** In the list of instructions of the grant proposal form: - under subheading "objective" is mentioned: "*provide a description of the target group (if there is one), categorizing relevant details by gender, age, cultural background and/or other relevant characteristics*". -under subheading"work plan" is mentioned: "*where a target group is involved, indicate how the factors of gender, age, cultural background and/or any other relevant characteristics that form an essential part of the objective will be addressed; you should also indicate the extent of any collaboration with the intermediate or ultimate target group (the point-of-view of patients/consumers)*"	**Programme Innovation ** There is no reference to sex or gender in the proposal form's list of instructions

There is no reference to sex or gender in the proposal form itself	There is no reference to sex or gender in the proposal form itself

We determined the number of proposals submitted to each programme which expressed the intention to take sex differences into consideration. We also identified those proposals in which no such intention was expressed, even though the topic of research indicated that it might have been relevant.

## Methods

The general procedure for applying for grants at ZonMw consists of three phases. First, applicants are asked to submit a brief summary of the study for which they are seeking grant support. These abridged proposals are reviewed by the ZonMw committee responsible for the programme in question. In the second phase, ZonMw invites a limited number of applicants to write a detailed grant proposal. In the third stage, following a peer review of these detailed proposals by experts in the field, the applicants are invited to respond to the reviewers' comments. On the basis of this response, the proposals are awarded their final grade. Those with the best grades are earmarked for grants [11,13,14]. The initial abridged proposals provide a good impression of the research questions that the applicants intend to answer, and of the methodology that they intend to use, prior to any interference from the peer reviewers. For this reason, we decided to use these initial proposals as the basis of our study.

### Characteristics of the programmes

We selected proposals that had been submitted to ZonMw's Prevention and Innovation programmes. Various characteristics of the programmes in question are described below.

Prevention is a four-year programme commissioned by the Ministry of Health. It has a total budget of € 48 million. Consisting of three sub-programmes, it provides grants for basic, applied, and implementation studies which focus on the primary and secondary prevention of medical conditions. The 2003 call for proposals listed the programme's priorities as chronic conditions, mental health, infectious diseases, and health promotion, as well as the methods and organization associated with health promotion strategies [13]. The purpose of the Innovation programme is to encourage innovative research on any theme by highly respected research groups [14]. This may include basic, clinical, population-based, or health systems research. The programme has an annual budget of 8 million euro [15]. See table 2 for examples of studies that have been approved for funding by these programmes.

**Table 2:** Examples of research proposals approved by the two programmes in question (randomly chosen) [16].

**Prevention**^**+**^	**Innovation**^**+**^
-Risk prediction based on vascular risk factors; Development of an instrument for guiding preventive measures in the elderly. -Rehabilitation and Sports: a study on the effect of a sports and physically active lifestyle stimulation programme during and after the regular rehabilitation treatment, on the degree of sports participation, daily physical activity and health status in a population of rehabilitating patients. -Development and evaluation of an educational intervention targeted at optimizing adherence to measures aimed at preventing asthma in children with a familial allergic disposition.	-Cellular reprogramming in neuromuscular tissues as a response to genetic lesions in the cellular network for energy homeostasis – Adaptation and (programmed) cell death. -Towards gene therapy for haemophilia: a research programme on the transfer of the genes for factor VIII and IX into hepatocytes and into the liver. -Functional and molecular characterization of the role of cytokines in development of human lymphocytes. -The role of the glycocalyx in myocardial perfusion and coronary endothelial function in health and disease

^+^As a result of privacy considerations, no titles of grant research proposals included in this study are shown.

### Data collection and analysis

We selected all 109 proposals submitted to the Innovation programme in 2003. The three sub-programmes of the Prevention programme received 313 proposals in 2003. Turboran, a randomization program, was used to randomly select 37% (115) of the proposals submitted to the three sub-programmes. The purpose was to obtain comparable numbers of proposals from both of the main programmes (Prevention and Innovation). Eleven proposals submitted to the Prevention programme were discarded as they were not available in digital form.

To determine how many research proposals expressed the intention to consider sex differences, a content analysis was conducted of all the selected proposals. To protect the anonymity of the applicants, only one researcher was allowed access to the full proposals and the content analysis had to be carried out at ZonMw's offices, under the supervision of a member of staff. DGK conducted the analysis. The aim was to search for references to an intention to study sex differences^1 ^in the aims and objectives sections, and in the work plan or the methodology sections. To this end the following steps were taken:

Firstly, the following data were recorded for all proposals: full title and the type of study: basic study, applied study, implementation study, and whether or not the study considered a non-sex-specific condition. Two of the proposals for applied studies in the Prevention Programme dealt with conditions that only occur in one sex (menopause, post-partum incontinence). These proposals were not included in the next step of content analysis, as we were only interested in studies of conditions that can occur in both sexes (further details of the definitions used and practices adopted are available from the corresponding author).

Secondly, all proposals that considered non-sex-specific conditions were read in full, with a focus on the following questions:

• Are sex differences considered in the aim of the study?

• Are sex differences considered in the objective of the study?

• Does the work plan give details of how sex differences will be analysed?

To double check the content analysis, a word scan was performed on the proposals using the following query: *sex*, *gender*, *male*, *female*, *men*, *women*, *boy*, *girl*.

All proposals that produced an affirmative answer to one of the three questions above were included in the category of proposals reflecting the intention to consider sex differences. The proposals in question were also coded according to the degree to which the intention to consider sex differences was fleshed out in the proposal:

• "Little intention": if one of the three above questions was answered affirmatively.

• "Marked intention": if two or three of the above questions were answered affirmatively.

Following the selection of those proposals which contained an intention to consider sex differences, two other researchers were asked to assess the remaining proposals, to determine in which cases it may have been relevant to focus on sex differences. Both of these researchers have broad expertise in biomedicine and public health.

To protect the applicants' privacy, our researchers were only allowed to screen the titles of the studies in question, rather than the proposals themselves. For that reason a document was created with a list of titles of those remaining proposals that did not reflect the intention to consider sex differences.

The researchers were asked to screen these titles individually and to assign them to the following broad categories:

• Consideration of sex differences *not to be expected*, given what is known about the topic of the study.

• Consideration of sex differences *could have been expected*, given what is known about the topic of the study.

The researchers were not given a rigorously fixed list to guide their decisions, because we assumed that their knowledge of the field would be sufficient to this end. The inter-rater agreement was measured using Cohen's kappa. The value obtained was 0.47. Where there was disagreement, the researchers sought to achieve consensus through discussion.

### Comparison of the programmes

Specific data on the total number of proposals that did or did not express the intention to consider sex differences is presented for each of the programmes. This data is classified according to study type (basic, applied, implementation).

The two programmes were compared on the proportion of proposals with a positive intention to consider sex differences. To determine whether the difference in proportions was significant, the 2-sided Fisher Exact test was used. For the difference in proportions 95% confidence intervals were calculated. Inclusion of the value zero in the interval indicates that the difference is not statistically significant at the 5% level. We expected the proportion of proposals with a positive intention to be lower in the Innovation programme than in the Prevention programme, because the latter programme had taken more explicit measures to encourage this. Because the intention to consider sex differences varied with study type and because the distribution of protocols across study type varied between the two programmes, proportions were also adjusted for study type and subsequently compared, using the Mantel-Haenszel test for stratified analysis. Computations were conducted using Stata 9.2. software (StataCorp, Texas, USA)

## Results

Of the total of 104 proposals received by the Prevention programme, we identified 24 proposals (23%) in which investigators expressed the intention to consider sex differences in their study's aims, objectives or work plan (see table 3). For seven of these proposals, this intention was fleshed out in the aim and/or objectives as well as in the work plan (classified as a "Marked intention"). Of the 109 research proposals that were submitted to the Innovation programme, 11 proposals (10%) were found to express an intention to consider sex differences. Of these, 3 actually gave specific details of this throughout the proposal. The two programmes differed in the composition according to study type. Whereas most of the studies submitted to the Prevention programme were either applied or implementation studies (93 out of 104), most of the studies submitted to the Innovation programme were basic studies (94 out of 109).

**Table 3:** Proposals in which the applicants expressed an intention to consider sex, subdivided into study type

**Intention to consider sex differences**	**Present**			**Absent**	**Number of proposals submitted**
	**All**	**Marked **	**Little**		
	N (%)	N (%)	N (%)	N (%)	N (%)
Programme Prevention	**24 (23%)**	**7 (7%)**	**17 (16%)**	**80 (77%)**	**104 (100%)**
Study type					
*basic*	3	2	1	4	7
*applied*	18	5	13	59	77
*implementation*	2	0	2	14	16
*classification unclear*	1	0	1	3	4
					
Programme Innovation	**11 (10%)**	**3 (3%)**	**8 (7%)**	**98 (90%)**	**109 (100%)**
Study type					
*basic*	9	3	6	85	94
*applied*	1	0	1	10	11
*implementation*	0	0	0	0	0
*classification unclear*	1	0	1	3	4

In the Prevention programme, 80 of the 104 proposals did not express an intention to focus on sex differences (see table 4). Two of these proposals dealt with conditions that only occur in one sex and were classified as consideration of sex differences "not to be expected". According to the opinion of the two experts, in 69 of those 80 proposals (66% of total) it may well have been relevant to do so. In the Innovation programme, 98 of the 109 proposals did not address sex differences. With respect to 22 of those 98 proposals (20% of total), the experts expressed the view that this might well have been relevant. If we combine the data regarding the types of study submitted to both programmes, table 4 shows that 54 of the 69 (78%) proposals for applied studies that did not intend to address sex differences could have been expected to do this according to the experts. This was the case for 19 of the 89 (21%) proposals for basic studies of this category and for 13 of the 14 (93%) proposals for implementation studies.

**Table 4:** Proposals in which the applicants did not express an intention to consider sex^+^, subdivided into study type.

**Intention to consider sex differences**	**Expected**	**Not expected**	**Total no intention**	**Number of proposals submitted**
	N (%)	N (%)	N (%)	N (%)
Programme Prevention	**69 (66%)**	**11 (11%)**	**80 (77%)**	**104 (100%)**
Study type				
*basic*	4	0	4	7
*applied*	48	10	59^++^	77
*implementation*	13	1	14	15
*classification unclear*	3	0	3	4
				
Programme Innovation	**22 (20%)**	**76 (70%)**	**98 (90%)**	**109 (100%)**
Study type				
*basic*	15	70	85	94
*applied*	6	4	10	11
*implementation*	0	0	0	0
*classification unclear*	1	2	3	4

^+ ^experts made an assessment by screening the title of a proposal

^++ ^includes one proposal on which the experts were indecisive whether attention to sex differences could have been relevant or not.

A comparison between the two grant programmes showed that the Prevention programme received a significantly higher proportion of proposals expressing an intention to consider sex differences than the Innovation programme (table 5). The difference between the two programmes was 13.0 % (95% CI, 3.1–22.9) when no adjustment is made for the differences with respect to the types of study that were submitted to the two programmes. The difference is 19.5% (95% CI, 1.4–37.6) when adjustments are made for study type. We did not find any significant differences between the two programmes for the different study types.

**Table 5:** Comparison of proposals with expressed intention to consider sex differences between the programmes Prevention and Innovation; overall, per study type and adjusted for study type.

	**Proposals expressing intention to consider sex differences**				**Difference between programmes**	
	Prevention (N = 104)		Innovation (N = 109)		Absolute	95% CI^+^
	%	N	%	N	%	
Overall ^(F)^	23%	24/104	10%	11/109	13.0	3.1–22.9*
						
Study type/difference corrected ^(F)^						
*basic*	43%	3/7	10%	9/94	33.3	-3.9–70.4
*applied*	23%	18/77	9%	1/11	14.3	-5.2–33.7
*implementation*	12%	2/16	0	0	-	-
*classification unclear*	25%	1/4	25%	1/4	0^++^	-60.0–60.0
						
Overall, adjusted for study type ^(M)^		22/88		11/109	19.5	1.4–37.6*

^+ ^CI: Confidence interval

^(F) ^2-sided Fisher Exact test

^(M) ^Mantel-Haenszel test. Study type 'implementation' is excluded from the stratified analysis because the type is not represented in the program Innovation.

* significant (p ≤ 0.05)

## Discussion

Towards the end of the 1990s, the Netherlands Organization for Health Research and Development was one of a group of international public organizations for the funding of health research that introduced the policy of encouraging health research workers to focus on diversity factors. The present study analysed proposals submitted to two important ZonMw grant programmes in 2003, to determine whether they gave any consideration to sex differences. Those that did not do so were subjected to a further examination by two experts to determine whether a consideration of these differences would have been relevant.

The Prevention programme involved a greater effort to alert applicants to ZonMw's diversity policy. In contrast to the Innovation programme, the instructions for applicants contained more specific references regarding diversity issues. This may account for the fact that, relative to the Innovation programme, the Prevention programme received a greater proportion of proposals (13%) expressing the intention to focus on sex differences. However, it should also be noted that in 66% of the proposals submitted to the Prevention programme that did not address sex differences, it might have been relevant to do so according to the experts. By contrast, this was only the case in 20% of the proposals submitted to the Innovation programme. This raises questions with regard to the effectiveness of the approach taken by the Prevention programme. We found that only 21 % of the proposals for basic studies submitted to both programmes that did not intend to address sex differences could have been expected to do this according to the experts, while this was the case for 78% of the proposals for applied studies and for 93% of the proposals for implementation studies. This suggest that the proposals for basic studies in our sample performed relatively well with respect to an expected consideration of sex differences. This finding is intriguing, as the general recognition that sex is an important variable that should be taken into account in basic research is a relatively recent development [17-20]. Further research is needed to determine specific barriers and facilitating factors for the consideration of sex differences in the design of applied, implementation and basic studies.

This study was subject to the following limitations. Firstly, it was based on the initial, abridged research proposals that were submitted to ZonMw. It could be argued that the full proposals which ZonMw receives in the later stages of the grant application cycle might have provided more information concerning the approach to sex differences adopted by the project in question. However, we believe that the initial proposals provide an accurate impression of the way in which the research community interprets ZonMw's diversity policy. Secondly, although our initial aim was to determine whether a given research proposal expressed an intention to consider sex and/or gender differences, this was found to be impossible in practice. The reason for this is that while these concepts are well defined in the literature [21-23], investigators still use them interchangeably when writing research proposals. Accordingly, we were unable to determine whether the proposed studies addressed sex differences, gender differences, or both. Thirdly, when assessing the proposals that did not focus on sex differences, to determine in which cases it may have been relevant to do so, our researchers were only allowed to screen the titles of the studies in question. This was done in order to protect the applicants' privacy. A full reading of the proposals may have led to somewhat different judgements. Furthermore, the two researchers did not screen the titles of the earlier selected group of proposals that reflected an intention to focus on sex differences. This might have been a useful check of their method for screening the interest of sex differences for the topic of the study, only on the basis of the available titles. Despite these limitations, it is our contention that the list of proposals selected by our researchers gives a good impression of those studies in which a consideration of sex differences may have been relevant.

Our study suggests that the way in which this ZonMw policy has been implemented, particularly in the Innovation programme, does not give applicants sufficient incentive to routinely consider sex differences when drafting their research proposals. ZonMw's diversity policy is very broad based. It is intended to encourage the inclusion of numerous diversity factors in a wide range of studies (basic, applied and intervention). By adopting an open approach of this kind, ZonMw has opted to grant applicants considerable freedom in selecting what they consider to be relevant to their requirements, and in discarding what is not. Indeed, a previous analysis of health proposals submitted to ZonMw concluded that some researchers tended to prefer a consideration of ethnic variations above sex or gender differences [24].

At international level, there is considerable variation in the policies that public organizations for the funding of health research employ in an attempt to heighten the focus on sex differences. For instance, unlike the ZonMw policy, the NIH policy is supported by legislation. Funding is conditional upon compliance, and the policies of the NIH, Health Canada and the EU are more tightly focussed on promoting a consideration of sex differences in particular [3,8,25-27]. These policies have also been more regularly monitored than the ZonMw policy. The monitoring in question is carried out either by the organizations themselves or by independent researchers. These studies showed that the inclusion of both sexes in a study does not automatically lead to the analysis and reporting of data by sex [4-7]. This, together with other results, has prompted these organizations to take further actions to enhance guidance. Indeed, for many researchers in health research, the idea that a consideration of sex or gender differences could be relevant to their research is still something of a novelty. As the NIH inclusion policy has shown, any attempt to encourage a greater focus on sex differences in research involves complex procedures and requires considerable endurance [4-6,28].

This study suggests that ZonMw's current diversity policy may be too broad, and that investigators need better guidance if they are to take sex differences into account in their research proposals. Therefore we formulated some recommendations.

Firstly, in order to help applicants understand what is expected from them, they must be supplied with a clear set of instructions. One example of clearly written instructions is the amended NIH Policy and Guidelines on the Inclusion of Women and Minorities as Subjects in Clinical Research [29]. Another is the Vademecum for Scientific Officers and Project Officers produced by the EU's Directorate General of Research [9].

Secondly, it should be made clear to applicants that the reviewers of their research proposals will be carrying out specific checks to determine whether sex differences have been appropriately addressed.

Thirdly, initiatives should be launched to provide appropriate training for the staff of organizations that fund health research, as well as for present and future applicants and reviewers. The training in question should address the relevance of focussing on sex differences in health research, giving examples of how this should be done in different types of studies (basic, applied, implementation). Here too, the materials produced by the NIH Office of Women's Health and the EU are useful examples [9,30].

Fourthly, as with any policy, progress should be monitored regularly. Furthermore, clear indicators are needed in order to measure progress.

## Conclusion

In conclusion, 35 of the 213 proposals submitted to two ZonMw programmes in 2003 expressed the intention to address sex differences. ZonMw's diversity policy may have had a role in this. The Prevention programme attracted more proposals paying attention to sex differences than the Innovation program. This may be due to the fact that the first programme provided more explicit instructions on how to address this topic to applicants. However, for 91 of the 178 proposals that indicated no intention to consider sex differences, it was evident from the subject matter in the proposals that such considerations may have been appropriate. Many of those proposals involve applied and implementation studies. The provisions of ZonMw's present policy need to be amended and there must be better monitoring, to ensure that sex differences are systematically addressed in the development of research proposals.

## Competing interests

The author(s) declare that they have no competing interests.

## Authors' contributions

JAH and NSK were involved in drawing up the research proposal, on the basis of which funding was provided. The data was collected and analysed by DGK, who was advised by NSK and an epidemiologist. DGK wrote the first drafts of the paper, which was then edited by JAH and NSK. JAH is the guarantor of the study. All authors read and approved the final manuscript.

## Appendix- Footnotes

^1^The terms sex and gender are often used interchangeably in the literature and by ZonMw, and in this article we use the term sex to refer to male/female differences, biological sex differences as well as socially constructed gender differences.
